# Distinctive cognitive phenotypes in Parkinson’s disease patients with GBA mutations and without dementia: a multicentre cross-sectional retrospective study

**DOI:** 10.1016/j.prdoa.2025.100365

**Published:** 2025-07-04

**Authors:** Chiara Longo, Marco Liccari, Ruggero Bacchin, Costanza Papagno, Donatella Ottaviani, Raffaella Di Giacopo, Mauro Catalan, Alina Menichelli, Massimo Marano, Alessio Di Fonzo, Giovanni Duro, Carmela Zizzo, Paolo Manganotti, Bruno Giometto, Maria Chiara Malaguti

**Affiliations:** a Department of Neurology, Azienda Provinciale per i Servizi Sanitari (APSS), 38122 Trento, Italy; b Department of Psychology, Azienda Provinciale per i Servizi Sanitari (APSS), 38122 Trento, Italy; c Clinical Unit of Neurology, Department of Medical, Surgical and Health Sciences, Cattinara University Hospital, Azienda Sanitaria Universitaria Giuliano-Isontina (ASUGI), Trieste, Italy; d Pineta del Carso, Viale Stazione, 26, 34011 Aurisina, Italy; e Center for Mind/Brain Sciences (CIMeC), University of Trento, 38068 Rovereto, Italy; f Unit of Neurology, Neurophysiology, Neurobiology and Psychiatry, Department of Medicine, University Campus Bio-Medico of Rome, Rome, Italy; g Fondazione Policlinico Universitario Campus Bio-Medico, Rome, Italy; h Dino Ferrari Center, Neuroscience Section, Department of Pathophysiology and Transplantation, University of Milan, Milan, Italy; i Foundation IRCCS Ca’ Granda Ospedale Maggiore Policlinico, Neurology Unit, Milan, Italy; j Institute for Biomedical Research and Innovation (IRIB), National Research Council (CNR), 90146 Palermo, Italy; k Department of Medicine, Surgery and Health Sciences, University of Trieste, Trieste, Italy

**Keywords:** Parkinson’s Disease, GBA mutation, Cognition, Mild Cognitive Impairment, Memory

## Abstract

**Background:**

Cognitive impairment is a major non-motor complication of Parkinson’s disease (PD). GBA mutations are associated with an increased risk, with PD-GBA+ patients typically showing earlier disease onset and faster cognitive decline. However, the specific cognitive phenotype of these patients remains unclear.

**Aim:**

To provide a detailed neuropsychological profile of PD-GBA+ patients compared to PD-GBA− patients.

**Methods:**

Data from 18 PD-GBA+ and 68 PD-GBA− patients were retrospectively analyzed. All participants underwent comprehensive neurological evaluations of motor and non-motor symptoms, along with a Level II neuropsychological assessment based on the MDS criteria for mild cognitive impairment (MCI). Patients with dementia were excluded.

**Results:**

PD-GBA+ patients showed significantly lower cognitive performance, particularly on the RAVLT immediate recall (RAVLT-IR, p < 0.001) and delayed recall (RAVLT-DR, p = 0.002). All PD-GBA+ patients exhibited an amnestic multi-domain MCI phenotype. In contrast, the PD-GBA− group predominantly showed a non-amnestic single-domain MCI, characterized by a dysexecutive profile. Additionally, PD-GBA+ patients had a higher prevalence of freezing of gait (p < 0.001), right-sided motor symptom lateralization (p = 0.011), and REM sleep behavior disorder (p = 0.006).

**Conclusions:**

PD-GBA+ patients exhibit a distinctive cognitive phenotype, already evident in the early stages of the disease. These results highlight the added value of Level II neuropsychological assessment in accurately characterizing the clinical phenotype and identifying patients at higher risk of developing dementia. Early cognitive profiling may thus contribute to more targeted monitoring and personalized therapeutic strategies.

## Highlights

• PD-GBA1 patients were compared with a clinically matched PD group.
• PD-GBA1 patients presented a distinctive amnestic multi-domain MCI phenotype.
• Cognitive profiling support clinical phenotype, prognosis, and personalized care.

## 1 Introduction

Cognitive impairment is among the most debilitating non-motor complications of Parkinson's disease (PD) [1] and can span along a continuum ranging from mild cognitive decline (PD-MCI) to overt dementia (PD-D). PD-MCI is characterized by cognitive impairments that do not interfere with daily activities [2], but represents a risk factor for dementia. A systematic review [3] identified advanced age, low education, long disease duration, anxiety, and depression as factors associated with PD-MCI, identifying the MCI multi-domain as the most frequent variant. Currently, the diagnosis of PD-MCI relies on the criteria established by the Movement Disorder Society (MDS) [2], which recommends the evaluation of five cognitive domains (attention and working memory, executive functions, episodic memory, language, and visuospatial abilities) by means of two levels of assessment: Level I, which includes a screening test or a single test for each cognitive domain; and Level II, which requires at least two tests for each cognitive domain. Several studies have demonstrated the superiority of Level II assessment over Level I in detecting MCI [2,4,5]. However, most studies still apply a Level I assessment, that could lead to an underestimation of the frequency of MCI preventing an accurate analysis of cognitive phenotypes.

In the context of cognition, the role of *GBA1* is particularly relevant in PD. Recent studies have reported mutations in the *GBA1* gene as one of the most significant genetic risk factors for PD [6,7]. However, these estimates largely derive from studies in European populations. As highlighted by recent global research efforts, the genetic architecture of PD can vary significantly across population [8,9]. Indeed, the more severe phenotype, has been suggested to be even more common in certain populations, particularly Asian ones [10,11].

Clinically, PD patients with GBA mutations often present cognitive and motor impairments at an earlier age of onset and experience a more rapid progression of the disease [6]. Additionally, they show a higher prevalence of axial motor involvement [6] and are more likely to experience non-motor symptoms, including cognitive impairment [12].

As already reported, *GBA1* mutation status may be an independent risk factor for cognitive impairment in PD patients, especially affecting memory and visuospatial domains [13]. Moreover, *GBA1* mutation carriers exhibited greater impairments in working memory, executive function, and visuospatial abilities than non-carriers [14]. However, in the two studies mentioned above [13,14], the average disease duration at the assessment was quite advanced (15 and 8 years). The presence of long-term disease could affect the cognitive data, as it may reflect not only the impact of the *GBA1* mutation but also the effect of the disease duration itself.

In this scenario, our study aims to deeply investigate the neuropsychological profiles of PD carriers of a *GBA1* mutation (PD-GBA+) versus non carriers (PD-GBA−) with a short disease duration through a comprehensive II Level neuropsychological assessment.

## 2 Methods

### 2.1 Participants

In this retrospective cross-sectional study, data were collected between 2022 and 2024 from the Movement Disorders Outpatient Clinic of the University Hospital of Trento and Trieste (Italy), and from the Centre of Cognitive Rehabilitation in Rovereto (Italy).

In each center, the same motor and clinical variables were collected, and a same protocol was used for cognitive assessment.

The study has been approved by the local ethics committee and informed consent was obtained from all patients.

### 2.2 Inclusion/exclusion criteria

Patients were diagnosed with PD based on Postuma et al. [15] criteria. All participants had a Montreal Cognitive Assessment (MoCA) score above sixteen [16] and completed a Level II cognitive assessment [2,5]. Only those classified as cognitively unimpaired (PD-CU) or with mild cognitive impairment (PD-MCI) [2,17] were included in the study. All patients were screened for *GBA1* mutations [7].

### 2.3 Neurological assessment

Motor evaluations included disease duration, motor phenotype (i.e., tremor-dominant, akinetic-rigid), most affected side (left or right), and standardized scales for motor symptoms and disease stage, such as the MDS-Unified Parkinson’s Disease Rating Scale Part III (UPDRS-III) and the Modified Hoehn & Yahr (H&Y) scale. Data on freezing of gait (FOG), motor fluctuations, and dyskinesias were also gathered as dichotomous variables (presence/absence).

Non-motor symptoms were thoroughly assessed, including REM sleep behaviour disorder (RBD), hallucinations, as well as autonomic dysfunction, covering orthostatic hypotension and urinary problems. Behavioural aspects, such as impulse control disorders (ICDs) were evaluated alongside information on the use of antidepressants and acetylcholinesterase inhibitors. For all the non-motor data, a dichotomous variable indicating absence/presence was collected.

### 2.4 Neuropsychological assessment

Patients in both groups underwent a Level II cognitive assessment [2], conducted during the ON phase. Specifically, patients were initially screened with the MoCA [18], and only those with scores >16 were included in the study [16]. The cognitive evaluation included several standardized tests, based on the evidence that this battery classified patients in PD-CU or PD-MCI with an accuracy of 90.6 % [5]. Specifically, attention and working memory were assessed using the Digit Span Backward, Attentional Matrices, and Trail Making Test. Memory was evaluated through Digit Span Forward, Corsi Block-Tapping Test, Rey Auditory Verbal Learning Test–Immediate and Delayed Recall, and Rey-Osterrieth Complex Figure–Delayed Recall. Executive functions were tested with the Stroop Test, Phonemic Verbal Fluency, and the Rey-Osterrieth Complex Figure–Copy. Language abilities were examined by means of Semantic Verbal Fluency, Object Picture Naming, and Action Naming. Visuospatial abilities were assessed with the Line Orientation Judgment test and the Unknown Face Recognition test. Finally, the Ekman Test was administered for social cognition assessment. Furthermore, anxiety and depression were assessed using the Geriatric Depression Scale (GDS) [19] and the Parkinson Anxiety Scale (PAS) [20]. The test references are available in Longo et al. [5].

As in Longo et al. [5], patients were classified as PD-CU or PD-MCI based on Italian normative cut-off scores and on MDS criteria, with the additional consideration of social cognition.

Across all participating centres, identical motor and clinical variables were collected and a uniform cognitive assessment protocol was applied. Genetic sampling and cognitive testing were both offered as part of each centre’s routine clinical practice.

### 2.5 Statistical analysis

Preliminary statistical analyses were performed to evaluate data distribution (Shapiro-Wilk test) and to compare demographic variables (i.e., age, education, and gender) between groups (GBA+, GBA-) through Independent Samples T-Test (U Mann-Whitney) or Chi-squared test.

Performances on cognitive and motor tests between PD-GBA+ and PD-GBA- were compared using parametric one-way variance analysis (ANOVA) or non-parametric analysis (Kruskal-Wallis). Post-hoc analyses were carried out through the Games-Howell test (for parametric statistics) and Dwass-Steel-Crotchlow-Fligner pairwise comparison (for non-parametric statistics). Since GBA+ patients tended to be younger than GBA– patients, we compared the two groups using adjusted scores. These adjustments follow Italian normative data [21] and included age, education, and, when necessary, gender. Statistical significance was set at <0.05. Statistical analyses were conducted using JASP software (version 17.2.1).

## 3 Results

A total of 405 PD patients were screened for the *GBA1* mutation. As shown in Supplementary Fig. 1, 43 patients tested positive: 25 were excluded due either to an incomplete Level II cognitive assessment (n = 18) or to a diagnosis of dementia (n = 7). Therefore, we included cognitive and motor data from 18 PD-GBA+ patients. The control group (PD-GBA-) included PD patients who tested negative for the GBA mutation and met the same inclusion/exclusion criteria of the PD-GBA+ group. The final dataset comprised 18 PD-GBA+ and 68 PD-GBA- patients. Refer to Supplementary Fig. 1 for further details regarding the patients selection process.

According to Clinvar (https://www.clinicalgenome.org) and to HGMD (www.hgmd.cf.ac.uk) databases [7], out of 18 patients with a positive GBA test, two presented a severe mutation (P182L, L444P), eight had a risk variant (E326K, T369M), two had a mild variant (N370S, G46E), and six had a variant of unknown significance (K13R, P245T, V460M, W148R, E329H, L51L) (see Supplementary Table 1).

Demographic comparisons revealed no statistically significant differences between groups across any of the examined variables (p > 0.05) (Table 1). A trend emerged for GBA+ patients to be younger than GBA− patients; however, this difference was not statistically significant.

**Table 1:** Demographic and clinical variables of the two groups.

Variable	Group	Mean (SD)	W	p-value
Gender (M/F)	GBA-	38/30	/	0.201
	GBA+	13/5		
Age (y)	GBA-	64.75 (7.87)	769.500	0.050
	GBA+	60.89 (7.90)		
Education (y)	GBA-	11.28 (4.06)	619.500	0.940
	GBA+	10.94 (3.54)		
LEDD (mg)	GBA-	525.51 (368.22)	522.500	0.345
	GBA+	618.17 (407.47)		
UPDRS-III	GBA-	24.10 (13.16)	631.500	0.840
	GBA+	23.44 (12.15)		
H&Y	GBA-	1.81 (0.72)	559.00	0.553
	GBA+	1.97 (0.81)		
Disease duration (months)	GBA-	72.37 (61.77)	523.500	0.349
	GBA+	74.00 (43.42)		

LEDD = Levodopa Equivalent Daily Dose; UPDRS-III = Unified Parkinson's Disease Rating Scale-part III; H&Y = Hoehn and Yahr scale.

**Fig. 1:**
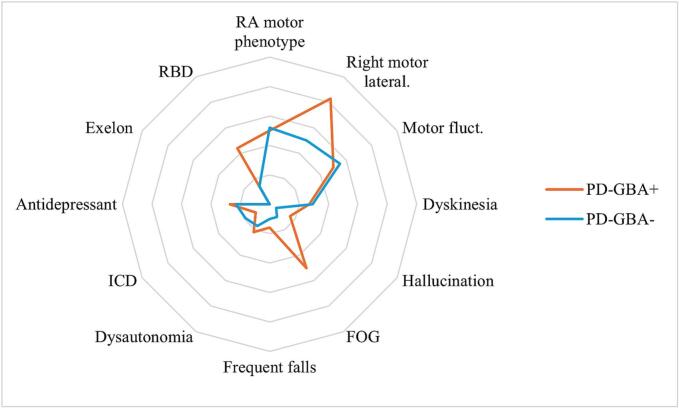
Radar graph of motor and non-motor symptoms in PD-GBA+ and PD-GBA-. The radar amplitude corresponds to the frequency of the disturbance. FOG = Freezing of gait; ICD = Impulsive control disorder; Motor fluct. = Motor fluctuations; RA motor phenotype = Rigid-akinetic motor phenotype; RBD = REM sleep Behavior Disorder; Right motor lateral. = Right motor lateralization.

Regarding motor and non-motor symptoms, significant group differences emerged for FOG, motor symptom lateralization, and REM sleep behaviour disorder (RBD). Specifically, PD-GBA+ patients were more likely to be right-lateralized (GBA+: 15/18, 83 % vs GBA-: 34/68, 50 %) and suffered higher rates of FOG (GBA+: 9/18, 50 % vs GBA-: 7/68, 10 %) and RBD symptoms (GBA+: 8/18, 44 % vs GBA-: 10/68, 14 %) than PD-GBA- (Fig. 1).

**Fig. 2:**
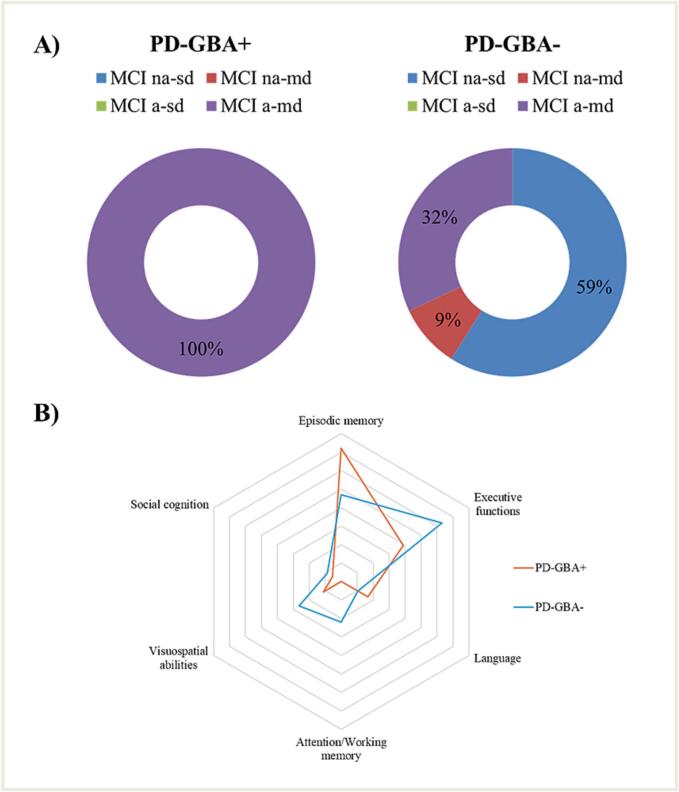
A) Types of MCI in the two groups. MCI a-sd = Mild Cognitive Impairment amnesic-single domain; MCI a-md = Mild Cognitive Impairment amnesic-multiple domain; MCI na-sd = Mild Cognitive Impairment non-amnesic-single domain; MCI na-md = Mild Cognitive Impairment non-amnesic-multiple domain. B) Radar graph of the frequency of cognitive domain compromised in each group.

As shown in Table 2, the comparison of the adjusted cognitive test scores revealed statistically significant differences between groups on the MoCA, RAVLT-IR, and RAVLT-DR tests (p < 0.05), with PD-GBA+ performing worse than PD-GBA-. No significant differences were found in visuospatial/visuoperceptual abilities (p > 0.05), language (p > 0.05), attention (p > 0.05), executive function (p > 0.05), and social cognition (p > 0.05). The prevalence of MCI was then assessed, showing that 38 % of PD-GBA+ patients met MCI criteria, compared to 32 % of PD-GBA- patients (p > 0.05). However, cognitive decline patterns varied between groups (Fig. 2): 13 of PD-GBA- patients (59 %) displayed a PD typical non-amnestic single-domain MCI (i.e., a dysexecutive syndrome), 7 patients (32 %) had amnestic multi-domain MCI, and two patients (9 %) exhibited non-amnestic multi-domain MCI. In contrast, all PD-GBA+ patients exhibited an amnestic multi-domain MCI phenotype.

**Table 2:** Performance on cognitive tests and emotional scales.

Cognitive test	Group	Mean (SD)	p-value
MoCA	GBA-	23.31 (3.56)	0.009*
	GBA+	21.97 (0.81)	
Digit Span Forward	GBA-	5.83 (1.09)	>0.05
	GBA+	5.78 (0.69)	
Digit Span Backward	GBA-	4.34 (0.94)	>0.05
	GBA+	4.37 (0.81)	
Corsi test	GBA-	5.13 (1.02)	>0.05
	GBA+	5.02 (0.79)	
Attentional Matrices	GBA-	47.54 (9.15)	>0.05
	GBA+	47.60 (7.18)	
RAVLT-IR	GBA-	44.91 (8.75)	<0.001**
	GBA+	35.47 (8.35)	
RAVLT-DR	GBA-	9.33 (3.03)	0.002*
	GBA+	6.75 (2.87)	
ROCF-C	GBA-	30.47 (5.38)	>0.05
	GBA+	31.19 (3.82)	
ROCF-DR	GBA-	16.06 (5.90)	>0.05
	GBA+	14.74 (6.60)	
Ekman test	GBA-	47.44 (7.54)	>0.05
	GBA+	48.87 (7.30)	
Stroop-errors	GBA-	1.69 (3.21)	>0.05
	GBA+	2.00 (3.27)	
Stroop-time	GBA-	22.53 (18.97)	>0.05
	GBA+	18.75 (9.61)	
TMT A	GBA-	41.19 (48.81)	>0.05
	GBA+	37.67 (15.09)	
TMT B	GBA-	102.45 (71.66)	>0.05
	GBA+	122.25 (58.66)	
TMT B-A	GBA-	72.34 (63.33)	>0.05
	GBA+	82.94 (47.34)	
Object naming	GBA-	46.63 (1.98)	>0.05
	GBA+	46.09 (2.45)	
Action naming	GBA-	46.55 (5.02)	>0.05
	GBA+	45.70 (3.83)	
Semantic fluency	GBA-	43.38 (12.74)	>0.05
	GBA+	40.35 (9.30)	
Phonemic fluency	GBA-	38.30 (13.69)	>0.05
	GBA+	34.16 (13.03)	
Line Orientation Judgment	GBA-	24.19 (5.28)	>0.05
	GBA+	25.22 (3.78)	
Face Recognition test	GBA-	44.60 (4.26)	>0.05
	GBA+	46.76 (2.99)	
GDS	GBA-	9.13 (7.43)	>0.05
	GBA+	9.88 (7.59)	
PAS	GBA-	13.56 (9.11)	>0.05
	GBA+	14.27 (6.62)	

GDS = Geriatric Depression Scale; MoCA = Montreal Cognitive Assessment; RAVLT-IR = Rey Auditory Verbal test–Immediate Recall; ROCF-C = Rey-Osterrieth Complex Figure–Copy; ROCF-DR = Rey-Osterrieth Complex Figure–Delayed Recall; Sem. Flu. = Semantic Fluency Test; PAS = Parkinson Anxiety Scale; TMT B-A = Trail Making Test Part B minus part A.

*p < 0.01, **p < 0.001.

Finally, no significant differences were found in anxiety and depression between PD-GBA+ and PD-GBA− patients (p > 0.05).

## 4 Discussion

Mutations in the *GBA1* gene are among the most prevalent genetic risk factors for PD, with severe variants associated with earlier symptom onset, faster disease progression, and greater cognitive decline [6,22]. Individuals carrying *GBA1* mutations exhibit an increased risk of developing dementia within the context of PD and Lewy Bodies Disease [10,23]. While research consistently shows that these mutations contribute to a higher susceptibility to cognitive impairment and dementia compared to non-carriers, the presence of MCI in the early stages of PD remains a topic of ongoing debate. Identifying the most common cognitive phenotypes can lead to targeted cognitive evaluations, enhance our understanding of the clinical landscape, and facilitate the early implementation of tailored care strategies.

In this context, our study aimed to comprehensively examine the cognitive phenotype of PD patients with GBA mutations in comparison to non-carriers, using an extensive II Level cognitive assessment. To better characterize the GBA1-PD phenotype, we also explored the presence of other clinical features.

After an average of six years of disease duration, our GBA+ cohort presented a lower overall cognitive functioning compared to GBA- as measured by means of the MoCA. This finding aligns with previous studies that relied solely on cognitive screening tests [24]. Although the MoCA is effective in identifying cognitive deficits in PD [2,5], it has limitations as a screening tool. Specifically, this test does not capture the full spectrum of cognitive alterations that may occur in these patients, nor does it provide a comprehensive understanding of their cognitive phenotype. Consequently, relying solely on the MoCA may overlook subtle cognitive impairments and nuances, highlighting the need for more thorough neuropsychological evaluations to accurately characterize the cognitive challenges faced by individuals with PD.

From the comprehensive Level II cognitive assessment in our sample, it emerges that GBA+ patients are more likely to develop episodic memory alterations, as shown by the RAVLT-IR and RAVLT-DR tests. This is particularly noteworthy given that the typical cognitive profile of PD patients is an attentional-dysexecutive one. Therefore, our results suggest that GBA+ patients exhibit a distinct cognitive pattern when compared to PD patients without GBA mutations, which has significant clinical implications, as the presence of episodic memory deficits are associated with an increased risk of developing dementia [25].

In our effort to assess the primary cognitive abilities affected in PD-GBA+ patients, we also aimed to determine whether these cognitive deficits were indicative of MCI. Using the MDS criteria [2], we evaluated the prevalence of MCI in both patients’ groups and found it to be similar between GBA+ and GBA- patients. This suggests that *GBA1* mutations do not significantly alter the overall likelihood of MCI, but affect the cognitive phenotype: while most GBA- patients exhibited the typical profile of single-domain non-amnestic MCI, primarily affecting executive functions, all GBA+ patients displayed the more severe multi-domain amnestic MCI. This pattern suggests that GBA+ patients are more susceptible to widespread cognitive impairment, which may increase their risk of functional decline.

Although our findings align with some studies [12], other research has identified deficits in visuospatial and visuoperceptual skills among GBA+ patients [13,14], that did not emerge in our sample. One possible explanation could be the differences in disease duration among the patient populations examined. Our sample had an average disease duration of 6 (±5) years, whereas Alcalay et al. [13] reported an average of 15.4 (±5.8) years, and Mata et al. [14] reported 8.4 (±5.7) years. Typically, visuospatial and visuoperceptual deficits in idiopathic PD are more common in the later stages, often associated with the onset of visual hallucinations [26,27]. Another possible explanation concerns the use of tests for each cognitive domain; this is an intrinsic limitation of neuropsychology in PD, as there are no clear guidelines on which tests to use for a specific cognitive function.

In our GBA+ cohort, we also noted a significantly higher prevalence of FOG and right-side lateralization at disease onset. Both axial disturbances and right-sided lateralization have been linked to an increased risk of cognitive decline [28,29]. These results suggest that the presence of FOG and right-sided motor symptoms may act as early indicators of cognitive vulnerability in GBA+ individuals, providing valuable insights for early non pharmacological intervention strategies.

Regarding non-motor symptoms, GBA+ individuals exhibited a higher incidence of RBD compared to GBA-, consistent with findings reported by Gan-Or et al. [30]. RBD is recognized not only as a potential early marker of neurodegenerative diseases but also as an indicator of an increased risk of cognitive decline [31]. Interestingly, previous research suggests that the most frequently reported subtype of MCI in patients with RBD is the non-amnestic type, whether single- or multi-domain, primarily characterized by deficits in attention and executive functions [32]. However, the limited number of patients with RBD in our sample prevents the possibility to confirm this observation.

Another limitation of this study is the heterogeneity in the severity of GBA mutations, which may have differentially influenced the cognitive outcomes observed. Due to the small sample size, stratified analyses were not feasible in our study, but future research should aim to better characterize clinical and cognitive phenotypes by differentiating according to mutation severity.

In conclusion, our study provides valuable insights into the cognitive profiles of PD-GBA+ patients without dementia, highlighting the distinct patterns and potential risk factors associated with these genetic mutations. The early presence of episodic memory impairment in GBA+ patients stands out as a critical marker, potentially signalling an elevated risk of dementia. This finding underscores the importance of early and comprehensive cognitive assessments to identify at-risk individuals and tailor intervention strategies accordingly.

Longitudinal studies, particularly with larger patient samples, will offer a clearer understanding of the specific cognitive phenotypes associated with *GBA1* mutations. This improved knowledge will enable clinicians to more accurately predict the progression of cognitive decline and tailor personalized care plans that encompass both pharmacological and non-pharmacological approaches.

## Funding sources

No specific funding was received for this work.

## CRediT authorship contribution statement

**Chiara Longo:** Writing – review & editing, Writing – original draft, Visualization, Project administration, Methodology, Investigation, Formal analysis, Data curation, Conceptualization. **Marco Liccari:** Writing – review & editing, Writing – original draft, Methodology, Data curation, Conceptualization. **Ruggero Bacchin:** Writing – review & editing, Methodology, Investigation. **Costanza Papagno:** Writing – review & editing, Investigation. **Donatella Ottaviani:** Writing – review & editing, Investigation. **Raffaella Di Giacopo:** Writing – review & editing, Investigation. **Mauro Catalan:** Writing – review & editing, Investigation. **Alina Menichelli:** Writing – review & editing, Investigation. **Massimo Marano:** Writing – review & editing, Investigation. **Alessio Di Fonzo:** Writing – review & editing, Investigation. **Giovanni Duro:** Writing – review & editing, Investigation. **Carmela Zizzo:** Writing – review & editing, Investigation. **Paolo Manganotti:** Writing – review & editing. **Bruno Giometto:** Writing – review & editing. **Maria Chiara Malaguti:** Writing – review & editing, Supervision, Methodology, Investigation, Conceptualization.

## Declaration of competing interest

The authors declare that they have no known competing financial interests or personal relationships that could have appeared to influence the work reported in this paper.
