# Supplementary material for: Distinctive cognitive phenotypes in Parkinson’s disease patients with GBA mutations and without dementia: a multicentre cross-sectional retrospective study
Source: Clin Park Relat Disord. 2025 Jul 4;13:100365. doi: 10.1016/j.prdoa.2025.100365 (PMC12274794; doi:10.1016/j.prdoa.2025.100365)
Supplement: Supplementary Data 1 [file mmc1.docx]

**Distinctive cognitive phenotypes in Parkinson’s Disease patients with GBA mutations and without dementia: a multicentre cross-sectional retrospective study**

**Supplementary Figure 1.** Patients’ selection and screening process.
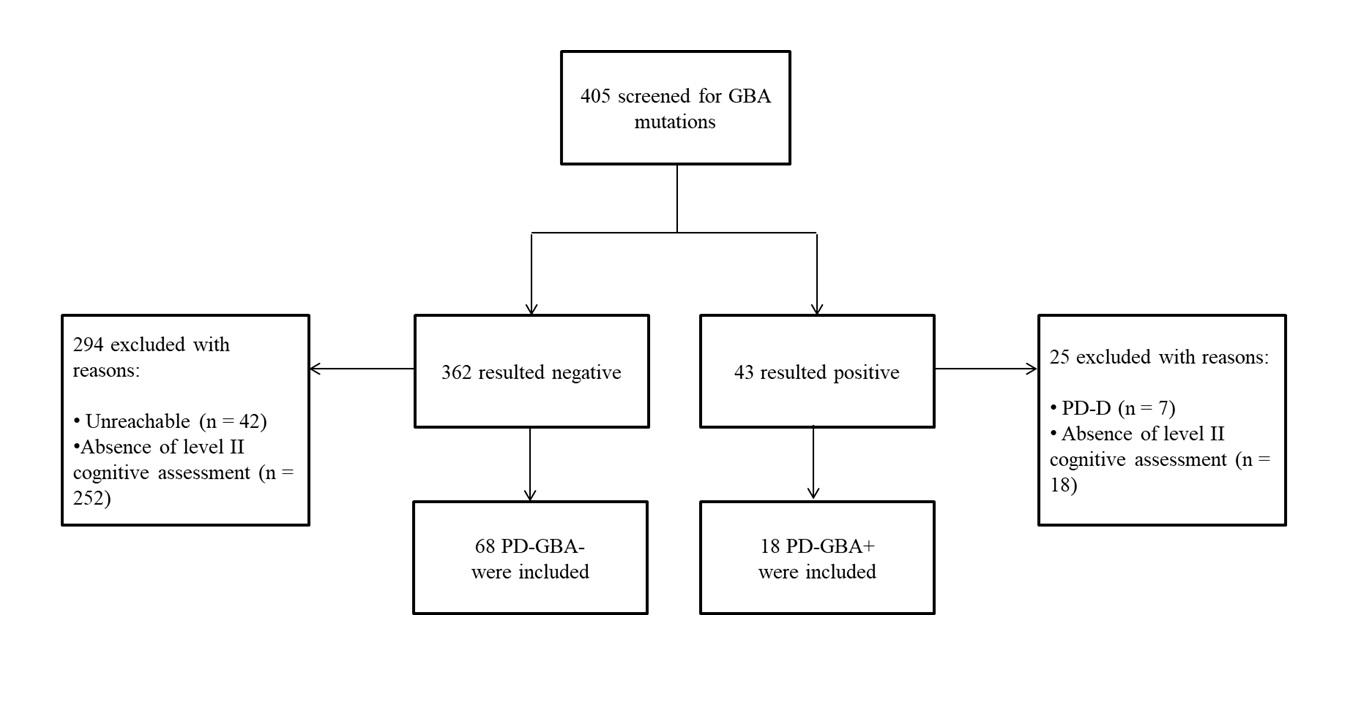


**Supplementary Table 1. *GBA1* mutations, severity, and significant clinical information.**

| **Patients code** | **GBA mutation** | **Mutation severity** | **Gender** | **Age** | **Cognition** | **Most affected side** | **FOG** | **RBD** |
| --- | --- | --- | --- | --- | --- | --- | --- | --- |
| #1 | K13R | Unknown | M | 43 | MCI na-md | Right | Yes | No |
| #2 | E326K | Risk variant | M | 60 | MCI na-md | Right | Yes | No |
| #3 | E326K | Risk variant | M | 62 | MCI na-md | Right | No | Yes |
| #4 | P182L | Severe | M | 48 | MCI na-md; More pronounced memory deficits. | Right | Yes | Yes |
| #5 | P245T | Unknown | M | 67 | MCI na-md | Right | Yes | No |
| #6 | V460M | Unknown | M | 61 | MCI na-md | Right | Yes | No |
| #7 | T369M | Risk variant | M | 64 | MCI na-md | Right | Yes | No |
| #8 | E326K | Risk variant | F | 67 | MCI na-md | Right | No | Yes |
| #9 | N370S | Mild | F | 57 | MCI na-md | Right | Yes | Yes |
| #10 | W148R | Unknown | F | 70 | MCI na-md | Right | No | No |
| #11 | L444P | Severe | M | 51 | MCI na-md; More pronounced memory deficits. | Right | No | No |
| #12 | G46E | Mild | M | 61 | MCI na-md | Right | Yes | No |
| #13 | E329H | Unknown | M | 56 | MCI na-md | Right | No | Yes |
| #14 | T369M | Risk variant | M | 64 | MCI na-md | Right | No | Yes |
| #15 | T369M | Risk variant | M | 69 | MCI na-md | Right | Yes | No |
| #16 | L51L | Unknown | F | 56 | MCI na-md | Right | No | Yes |
| #17 | T369M | Risk variant | M | 71 | MCI na-md | Right | No | No |
| #18 | T369M | Risk variant | F | 69 | MCI na-md | Right | No | Yes |

*F=female; M=Male; MCI na-md=Mild Cognitive Impairment-non amnestic multi-domain.*

**Supplementary Table 2. GBA1 mutations, disease severity, and relevant clinical information for the patients excluded due to PDD.**

| **Patients code** | **GBA mutation** | **Mutation severity** | **Gender** | **Age** | **Cognition** | **Most affected side** | **FOG** | **RBD** |
| --- | --- | --- | --- | --- | --- | --- | --- | --- |
| #1 | N370S | Mild | M | 78 | PD-D | Left | No | No |
| #2 | G64D | Severe | F | 72 | PD-D | Right | Yes | No |
| #3 | E326K | Risk variant | M | 73 | PD-D | Right | Yes | Yes |
| #4 | W148R | Unknown | M | 66 | PD-D | Right | No | No |
| #5 | T369M | Risk variant | F | 88 | PD-D | Right | Yes | Na |
| #6 | G64D | Severe | M | 75 | PD-D | Right | Yes | Yes |
| #7 | E326K | Risk variant | F | 68 | PD-D | Right | Yes | Yes |

*F=Female; M=Male; Na=Not available; PD-D=Parkinson’s Disease-Dementia..*
